# Altered Regulation of ELAVL1/HuR in HLA-B27–Expressing U937 Monocytic Cells

**DOI:** 10.1371/journal.pone.0070377

**Published:** 2013-07-22

**Authors:** Anna S. Sahlberg, Marja Ruuska, Kaisa Granfors, Markus A. Penttinen

**Affiliations:** 1 Department of Medical Microbiology and Immunology, University of Turku, Turku, Finland; 2 Department of Infectious Disease Surveillance and Control, National Institute for Health and Welfare, Turku, Finland; Virgen Macarena University Hospital, School of Medicine, Spain

## Abstract

**Objective:**

To investigate the role of HLA-B27 expression in the regulation of RNA binding protein (RBP) Embryonic Lethal Abnormal Vision (ELAV) L1/Human antigen R (HuR) expression in *Salmonella*-infected or LPS-stimulated human monocytic cells, since HuR is a critical regulator of the post-transcriptional fate of many genes (e.g. TNFα) important in inflammatory response.

**Methods:**

U937 monocytic cells were stably transfected with pSV2neo resistant vector (mock), wild type HLA–B27, or mutated HLA–B27 with amino acid substitutions in the B pocket. Cells were differentiated, infected with *Salmonella enteritidis* or stimulated with lipopolysaccharide. The expression levels of HuR protein and cleavage products (CP1 and CP2) were detected by Western blotting and flow cytometry. Specific inhibitors were used to study the role of PKR and p38 in HuR expression and generation of CPs. TNFα and IL-10 secretion after p38 and PKR inhibition were measured by ELISA.

**Results:**

Full length HuR is overexpressed and HuR cleavage is disturbed in U937 monocytic cells expressing HLA-B27 heavy chains (HC). Increased full length HuR expression, disturbed cleavage and reduced dependence on PKR after infection correlate with the expression of glutamic acid 45 in the B pocket that is linked to the misfolding of HLA-B27.

**Conclusion:**

Results show that the expression of HLA-B27 HCs modulates the intracellular environment of U937 monocyte/macrophages by altering HuR regulation. This phenomenon is at least partly dependent on the misfolding feature of the B27 molecule. Since HuR is an important regulator of multiple genes involved in inflammatory response observations offer an explanation how HLA-B27 may modulate inflammatory response.

## Introduction

Reactive arthritis (ReA) is a systemic inflammatory disease which belongs to a group of spondyloarthropathies (SpA). ReA is triggered by an infection with certain intracellular and gram negative bacteria like *Salmonellae, Yersiniae* and *Chlamydiae*. The mechanism for emergence of ReA remains still unclear but most likely both the triggering bacteria and host responses contribute. It is known that immune response to microbial infection is dependent on the characteristics of the host cell. For instance, it is suggested that the expression of HLA-B27, a known risk factor for ReA and all SpAs, modifies the intracellular environment of U937 monocyte/macrophages by altering important cellular signaling pathways [1]–[3]. This is of interest because the disease-triggering bacteria or bacterial antigens e.g. lipopolysaccharide (LPS) are shown to persist in ReA patients for an abnormally long time [4]–[7]. In fact, it is suggested that *Salmonella* is able to regulate its intracellular growth in the HLA-B27-positive cells and that might be a strategy for bacterial persistence [8]. Thus these observations suggest that the interaction between HLA-B27-expressing host cells and ReA-triggering bacteria is abnormal and leads to the persistence of the causative microbes/microbial compartments in ReA patients and to prolonged immune reaction. The mechanism by which HLA-B27 directly effects on this interaction and disease susceptibility has remained unclear but the unusual tendency of HLA-B27 heavy chains (HCs) to misfold and form aberrant dimers may play an important role [9]. HLA-B27 HCs peptide-binding groove, B pocket, has an amino acid composition that is conserved among disease-associated subtypes [10]. Particularly glutamic acid at position 45 (E45) and cysteine at position 67 (C67), seem to influence to the folding rate and dimer formation [11], [12]. Interestingly, altered intracellular signaling observed in HLA-B27–expressing U937 cells has been linked to E45 [1]–[3].

Gene regulation allows the cell to react and adapt to both internal and external challenges. Produced RNA transcripts are translated into proteins and in order to do that, the transcripts need to be protected from degradation and also be transported to a different location. The fate of the transcripts is guided by RNA binding proteins (RBPs). These molecules are essential in the maturation and function of mRNAs and they control processes like splicing, polyadenylation, nuclear degradation or export, localization, storage or degradation in cytoplasm and translation [13]. RBPs are in fact important regulators of cellular signaling and cell fate for stress-sensitive genes controlled by them play critical roles in mediating inflammatory responses. During stress reactions, such as activation of the inflammatory response, many cellular activities are interrupted. However, some molecules and mRNAs are conserved and production of factors important in stress response is initiated. In normal conditions, mRNAs containing AU-rich element (ARE) are typically short-lived but in cellular stress, mRNAs are stabilized and can be translated. In order to preserve ARE-containing mRNAs and guarantee the production of factors needed during stress response, mRNA stabilizing RBPs are needed [14].

A ubiquitously expressed RNA binding protein (RBP) Embryonic Lethal Abnormal Vision (ELAV) L1/Human antigen R (HuR) plays an important role in inflammatory and cellular stress responses [15] as it is a regulator of the post-transcriptional fate of ARE-containing mRNAs. For example, HuR regulates directly the fate of TNFα mRNA [16] and thereby HuR plays a major role in inflammatory disease. In fact, HuR can act both as a promoter and a suppressor of inflammation [17]. One other ligand mRNA for HuR binding is the CCAAT/enhancer-binding protein beta (C/EBPβ) [18]. Previously, we have detected an altered C/EBPβ expression pattern in human monocytic U937 cells expressing HLA-B27 [3]. Moreover, intracellular trafficking of many mRNA stability regulating factors is regulated by some major signaling pathways, including the mitogen-activated protein kinase (MAPK) cascade [19]. Activated MAPKs, including p38, may regulate the nucleocytoplasmic shuttling of HuR, and thus induce the cytoplasmic accumulation of HuR [20]. During early stress responses, HuR has an anti-apoptotic function but when cell death is inevitable, it has a pro-apoptotic role. This function appears to be regulated by caspase-dependent cleavage of HuR [13], [21]. HuR is mainly localized in the nucleus but can shuttle between nucleus and cytoplasm [22]. In cytoplasm, HuR can be cleaved to two cleavage products (CPs), HuR-CP1 (24 kDa) and HuR-CP2 (8 kDa), that have been linked to promotion of apoptosis [21]. Intriguingly, the cleavage of HuR is shown to be protein kinase RNA (PKR)-dependent [23] and we have observed increased PKR expression but decreased activation by phosphorylation in human monocytic U937 cells expressing HLA-B27 [3]. In these cells, also p38 MAPK pathway may be dysregulated [2]. For cellular signaling molecules found to be altered in HLA-B27–expressing U937 monocytic cells are regulated by HuR, we hypothesized that HuR expression might also be modified. This idea prompted us to study HuR expression and cleavage and whether these features are PKR- or p38 -dependent. To address the immunological relevance of PKR and p38 activity in HLA-B27–transfected U937 cells, we measured TNFα and IL-10 secretion.

## Materials and Methods

### Cell Lines and Transfections

The human monocytic cell line U937 was obtained from American Type Culture Collection (ATCC; Rockville, MD). It expresses HLA class I alleles A3, A26, B18, B51, Cw1, and Cw3 [24]. The U937 cells were cotransfected with HLA-B*2705 genomic DNA (B27g) [25], or mutant forms of HLA-B*2705 constructed by site-directed mutagenesis (Altered Sites; Promega, Madison, WI) [26] and plasmid pSV2neo (to confer resistance to Geneticin G-418) as described previously [27]. B27.H9F and B27.E45M have one amino acid substitution (F for H at position 9 and M for E at position 45). For mock transfection, cells were transfected with pSV2neo alone. Stable transfectants were selected for G-418 resistance (0.5 mg/ml) and for cell surface expression of the transfected molecule, as described previously [26], [27].

The transfectants were maintained in RPMI 1640 medium supplemented with 10% fetal bovine serum (FBS; PAA Laboratories, Linz, Austria); 1.8 mM L-glutamine (Cambrex, Belgium), and 50 µg/ml Gentamicin (Biological Industries, Kibbutz Beit Herennek, Israel) at 37°C in a humidified 5% CO_2_ atmosphere. New batches of the transfectants (stored at −135°C in Biofreezer) were introduced every three months. Before performing any experiments, the cells were tested for cell surface expression of the transfected molecules and also screened to exclude mycoplasma infection.

### LPS Stimulation

Cells were diluted to a concentration of 1.0×10^6^/ml and seeded into tissue culture flasks (25 cm^2^; Greiner, Frickenhausen, Germany) or 24-well plates (1.9 cm^2^; Greiner). Cells were differentiated to adherent macrophages with 10 ng/ml phorbol myristate acetate (PMA; Sigma Aldrich, St. Louis, MO) for 22–24 hours at 37°C in supplemented RPMI 1640 (described above). For LPS stimulation, 500 ng/ml of *S enteritidis* LPS (Sigma), was added. Inhibitors (described below) were added 15 minutes prior LPS stimulation.

### Infection with *S enteritidis*

Cells were diluted, seeded into culture flasks, and PMA-stimulated as described above. Before infection, the cells were washed with Hank´s balanced salt solution (HBSS) to remove non-adherent cells, the medium was changed to prewarmed RPMI 1640 supplemented with 10% human AB serum (Finnish Red Cross, Helsinki, Finland), and incubated for one hour. *S enteritidis* strain was a stool isolate from a patient with *Salmonella*-triggered ReA [27]. The cells were cocultured with *S enteritidis* for one hour. The extracellular bacteria were removed by washing three times with HBSS, and the incubation medium was changed to supplemented RPMI 1640 (described above) containing Gentamicin to kill the remaining extracellular bacteria. At this point, inhibitors were added as described below. Before infection, the bacteria were grown 18 hours at 37°C in 10 ml Luria-Bertani (LB) broth, then 500 µl of bacterial culture was transferred into fresh 10 ml LB for two hours to obtain the logarithmic phase of growth [27].

### Inhibition Assays

The inhibitors used; PKR inhibitor PKR+, control for PKR inhibition PKR-, p38 MAPK inhibitor SB202190, control for p38 MAPK infection SB202474, Raf1 kinase inhibitor I GW5074 (Raf), Casein kinase I inhibitor V (Cas) and Src kinase inhibitor I (Src; all Calbiochem, Darmstadt, Germany), were added to the culture flasks or plates (final concentration 10 µM) 1 hour after *S enteritidis* infection (after removing of the excess bacteria) or 15 minutes prior LPS stimulation. P38 MAPK inhibitor BIRB 796 (Calbiochem) was added to the culture flasks 15, 30, 60 or 90 minutes prior to stimulation.

### Preparation of Cell Extracts

Cells were harvested by scraping at indicated time points, washed twice with ice-cold phosphate buffered saline (PBS) and then immediately frozen at −70°C. Samples were resuspended with lysis buffer C (420 mM NaCl; 25% glycerol; 0,2 mM EDTA; 1,5 mM MgCl_2_; 20 mM HEPES pH 7,9; 0,5 mM DTT; and 0,5 mM PMSF pH 7,4), where Complete Mini Protease inhibitor cocktail tablet (Roche Diagnostics, Mannheim, Germany) and phosSTOP phosphatase inhibitor tablet (Roche) were added. Samples were incubated 60 minutes on ice at +4°C and centrifuged 20 minutes at 12 000 g at +4°C. Supernatants were collected as whole cell extracts (WCE) containing soluble proteins. The protein concentration was measured by Bradford protein assay (Bio-Rad, Hercules, CA) [3].

### Gel Electrophoresis and Western Blot Analysis

WCE (containing 30 µg of protein) in Laemmli buffer were separated on a 10% sodium dodecyl sulfate-polyacrylamide gel electrophoresis (SDS-PAGE) and transferred to nitrocellulose membrane (Protran Nitrocellulose; Schleicher & Schuell, Keene, NH) by semidry transfer apparatus (Bio-Rad). The blots were analyzed by enhanced chemiluminescence method (ECL; Millipore, Billerica, MA). Antibodies used: anti-HuR (6A97) 1∶400 (sc-71290, Santa-Cruz Biotechnology Inc., Santa Cruz, CA), anti-Hsc70 for HSPA8 1:10000 (Stressgen Bioreagents, Ann Arbor, MI), monoclonal anti-rabbit 1∶10000 (Dako, Glostrup, Denmark), anti-mouse (HRP) 1∶10000 (Dako) and anti-rat HRP 1∶10000 (Stressgen). Densitometric analysis was performed with MCID 5+ image analysis software. Intensity of each band was determined and proportioned to a loading control. The results are shown as a fold induction of relative intensity (RI) where the intensity of selected band was given value one, and the other intensities displayed in the same figure are proportional to that [3].

### Immunofluorescence Staining and Flow Cytometry

The cell surface expression of the transfected molecules was confirmed by immunofluorescence and flow cytometry (BD Immunocytometry Systems, San Jose, CA) as described previously [26], [27]. Briefly, cells were stained with anti-human HLA–B27 monoclonal antibody (mAb) (clone FD705-9EIEI0; One Lambda, Canoga Park, CA) and with fluorescein isothiocyanate–conjugated secondary antibody. Subclass-matched monoclonal antibody recognizing chicken T cells (3G6) was used as a negative control [26], [27].

For HuR analysis, PMA-maturated, LPS-stimulated and inhibitor-treated cells were collected at indicated time points and washed with PBS. Cells were fixed with 1.5% formaldehyde solution for 10 minutes at RT, and permeabilized with 100% ice cold methanol for 10 minutes. Cells were stored at −20°C in 100% methanol until staining. For staining, cells were divided in tubes (500 000 cells/tube) and washed twice with staining buffer (PBS supplemented with 1% BSA). Anti-HuR (6A97) antibody (sc-71290, Santa-Cruz Biotechnology Inc.) was added on cells (1∶50, 100 µl/tube) and incubated 30 minutes at RT in dark. Cells were washed three times with staining buffer. FITC-conjugated secondary antibody was added (1∶200, 100 µl/tube) and incubated 30 minutes at RT in dark. Cells were washed three times with PBS and 500 µl of EPICS-PBS (145 mM NaCl, 18 mM K_2_HPO_4_, 10 mM KH_2_PO_4_) was added on each tube. Analysis was performed by FACSCalibur flow cytometry (BD Immunocytometry Systems). The results are shown as a fold induction of relative amount of positive cells or fluorescence intensity where the value obtained at 0 hour time point was normalized to one, and the values at 5 hour time point are proportional to that.

### Quantitation of TNFα and IL-10 Secretion

For enzyme-linked immunosorbent assay (ELISA), the cells were seeded into 24-well tissue culture-plate and treated as described above. After 22–24 hour of incubation, fresh supplemented RPMI 1640 medium was dispensed on the cells. The cell free supernatants were collected after 6 h and 24 h incubation and frozen immediately −70°C before use. The amount of produced cytokine was measured by sandwich-ELISA method. Commercially available antibody pair Mab1 and Mab11 (551220 and 554511; BD Biosciences, San Jose, CA) was used to detect TNFα and JES3-9D7 with JES3-12G8 to detect IL-10 (554497 and 554499; BD Biosciences). Recombinant human TNFα and IL-10 (R&D Systems Inc., Minneapolis, MN) were used as a standard. Streptavidin–horseradish peroxidase (HRP) conjugate was purchased from Invitrogen and tetramethylbenzidine (TMB) substrate (Sigma-Aldrich). The absorbance was measured at a wavelength of 450 nm.

### Statistical Analysis

Statistical comparison of the data was performed with Wilcoxon matched pairs Signed-Rank and Wilcoxon Rank-Sum tests.

## Results

### Cell Surface Expression of the Transfected Class I MHC Molecules

U937 monocytic cells were stably transfected with genomic clone of HLA-B27 (B27g), different mutated forms of HLA-B27 (B27.E45M, and B27.H9F) and pSV2neo vector, or were mock-transfected with the vector only. The surface expression of the transfected molecules was always analyzed in the new batches of the cell lines (described previously) [26], [27]. As detected by flow cytometry, B27 molecules were expressed on cell surface nearly equally when compared to each other (results described previously) [26], [27]. The expression levels of transfected HLA-B27 on U937 cells were close to that physiologically expressed on the cell surface of peripheral blood monocytes [27].

### Altered Expression Profile of different Forms of HuR – Correlation with Glutamic Acid at Position 45 in the B Pocket of HLA-B27

We have previously shown that the expression of HLA-B27 influences on the intracellular environment of the monocytic U937 cells by altering the expression and activity of PKR and p38-dependent signaling [1]–[3]. Here we investigated whether HuR regulation is modulated in HLA-B27 expressing cells, since both PKR and p38 have been reported to participate in HuR regulation. In addition, we aimed to study whether the amino acid composition of HLA-B27 B-pocket, linked to the misfolding feature of the HLA-B27 molecule, would influence modulatory effects caused by B27 expression. Thus transfectants expressing mutated HLA-B27 HCs (H9F; F for H at position 9, misfolding, E45M; M for E at position 45, non-misfolding mutant) were also studied in all experiments.

**Figure 1:**
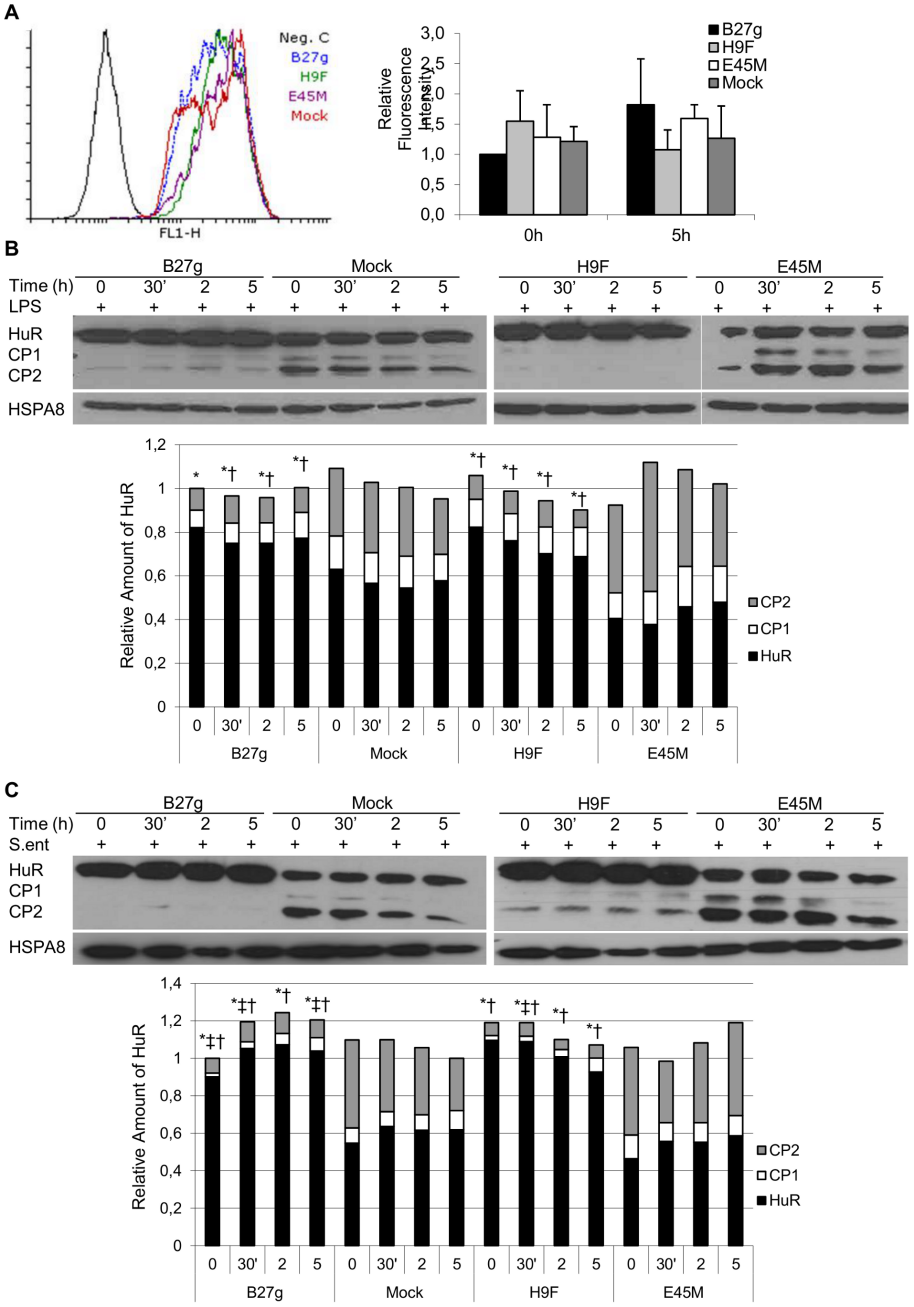
HuR expression and proportion of full length HuR and cleavage products in monocytic U937 cells. Full length HuR (36 kDa) and HuR cleavage products (CP1; 24 kDa and CP2; 8 kDa) in LPS-stimulated (A and B) and *Salmonella enteritidis*-infected (C) cells. The cells were transfected with vector (Mock) or genomic clone of HLA-B27 (B27g) or the HLA-B27 mutants B27.E45M (E45M) and B27.H9F (H9F). In all infection experiments, time ‘0′ refers to the time point where cells are infected with bacteria, incubated for 1 h and washed to remove the extracellular bacteria. In LPS experiments, time ‘0′ refers to the time of LPS stimulation. Expression of HuR was determined by FACS analysis (A) and SDS-PAGE and Western blotting (B, C). In A, a representative data is shown at 5 h time point together with analysis of a total of four independent experiments. In B and C, heat shock 70 kDa protein 8, HSPA8 was used as a loading control. Results from densitometric analysis of five independent experiments are shown in diagram. See Materials and Methods for details. * = P-value <0.05 for HuR, ^†^ = P-value <0.05 for CP2 and ^‡^ = P-value <0.06 for CP1 versus Mock at the respective time point.

First, we studied HuR expression with flow cytometry and FACS analysis. As shown in Fig. 1A, these experiments suggested that HuR is expressed similarly in all cell lines studied prior to the stimulation (0 h). Furthermore, no increase in the expression of HuR was observed 5 h after LPS stimulation. Next, we performed studies with Western blot to reveal the expression of HuR in more detail. In contrast to flow cytometry, studies with Western blot showed that full length HuR (36 kDa) is expressed in a higher level prior to LPS stimulation in cells expressing misfolding HLA-B27 HCs (B27g and H9F) when compared to Mock and E45M, a non-misfolding mutant of HLA-B27 HCs (Fig. 1B). Interestingly, also HuR CPs (CP1; 24 kDa and CP2; 8 kDa) were detected. Furthermore, we observed that the generation of CPs is decreased in B27g and H9F cells. Similar results were obtained when *Salmonella*-infected cells were studied (Fig. 1D and E). Neither LPS stimulation nor *Salmonella* infection influenced HuR expression in any of the cell lines studied within 5 hours of stimulation/infection.

In conclusion, western blot studies revealed that not only HuR (36 kDa) molecule is expressed but also HuR cleavage products (24 kDa and 8 kDa) are generated (Fig. 1 B–E). Full length HuR expression is increased in HLA-B27–expressing cells (P-value <0.05) and it is dependent on the glutamic acid 45 in B pocket of HLA-B27 molecule. A significant difference was observed between the transfectants in the generation of cleavage products even prior to LPS stimulation. In mock and E45M cells but not in B27g and H9F cells, HuR is strongly cleaved especially to CP2 (P-value <0.05 when B27g or H9F is compared to Mock). In B27g- and H9F–transfected cells, the amount of CPs is low but in Mock and E45M cells it is around the half of the total amount of HuR. No significant alteration in the expression level of any forms of HuR was observed in any of the cell lines studied after stimuli (stimulation with LPS in Fig. 1B, infection with *S enteritidis* in Fig. 1C).

### PKR Inhibition has only a Minor Influence on Full Length HuR Expression in HLA-B27-transfected Cells after Infection with *Salmonella*

**Figure 2:**
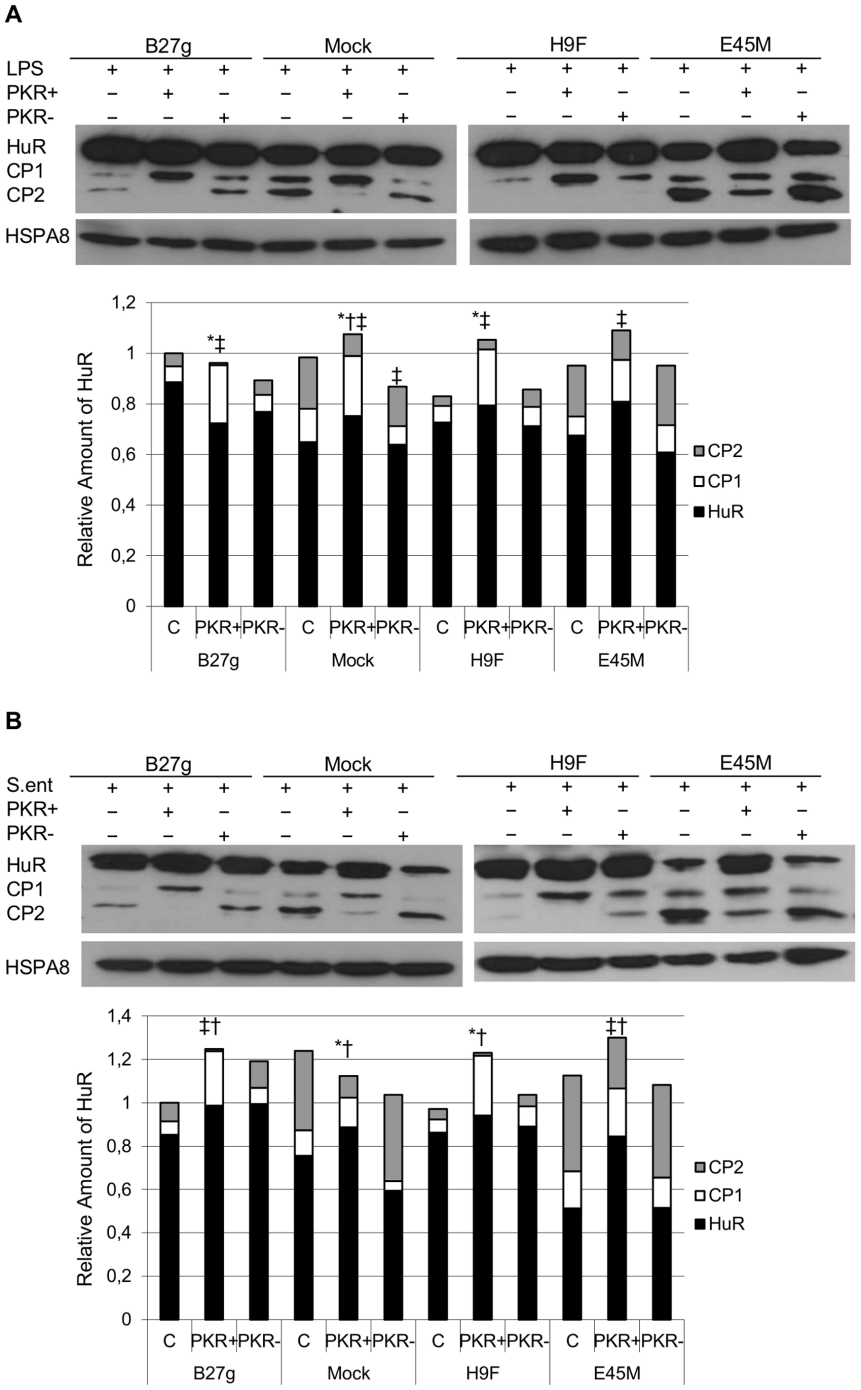
PKR inhibition effects on HuR expression and regulation in U937 monocytic cells. Full length HuR (36 kDa) and HuR cleavage products (CP1; 24 kDa and CP2; 8 kDa) in LPS-stimulated (A) and *Salmonella enteritidis*-infected (B) PKR inhibitor (PKR+)- or negative control for PKR inhibition (PKR-)-treated U937 Mock, B27g, H9F and E45M cells at 5 h time point. Expression of HuR was determined by SDS-PAGE and Western blotting. HSPA8 was used as a loading control. Results from densitometric analysis of five independent experiments are shown in diagram. See Materials and Methods for details. * = P-value ≤0.06 for HuR, ^‡^ = P-value ≤0.06 for CP1 and ^†^ = P-value ≤0.06 for CP2 versus untreated sample at the respective cell line.

Generation of HuR CPs has been reported to be PKR-dependent [23]. To investigate the relation between PKR and HuR in U937 transfectants, we inhibited the cells with a specific PKR inhibitor (PKR+). In order to ensure the specificity of the inhibitor, a control for PKR inhibitor was also used (PKR-, apart from PKR inhibition, the control compound has the same influence on cell behavior as the actual inhibitor). As shown in Fig. 2A and B, the inhibition of PKR influenced the expression level of different forms of HuR in all transfectants. In PKR-inhibited mock, E45M and H9F transfectants increased levels of full length HuR were observed 5 h after LPS stimulation (Fig. 2A). In contrast, PKR inhibition had only a minor influence on full length HuR expression in B27g transfectants. Level of CP1 was increased also in all transfectants after LPS-stimulation (P-value *≤*0.06, Fig. 2A). Interestingly, in *Salmonella* –infected mock and E45M cells, over one fourth of an increase in full length HuR expression was observed after PKR inhibition (Fig. 2B). In contrast, PKR inhibition had only a minor effect on full length HuR expression in B27g and H9F cells even after infection. In line with the results from LPS-stimulated cells, PKR inhibition influenced on the generation of CPs in all transfectants studied (P-value ≤0.06, Fig. 2B).

### p38 Inhibitor SB202190 Fails to Influence on HuR Expression in HLA-B27 Expressing Cells

**Figure 3:**
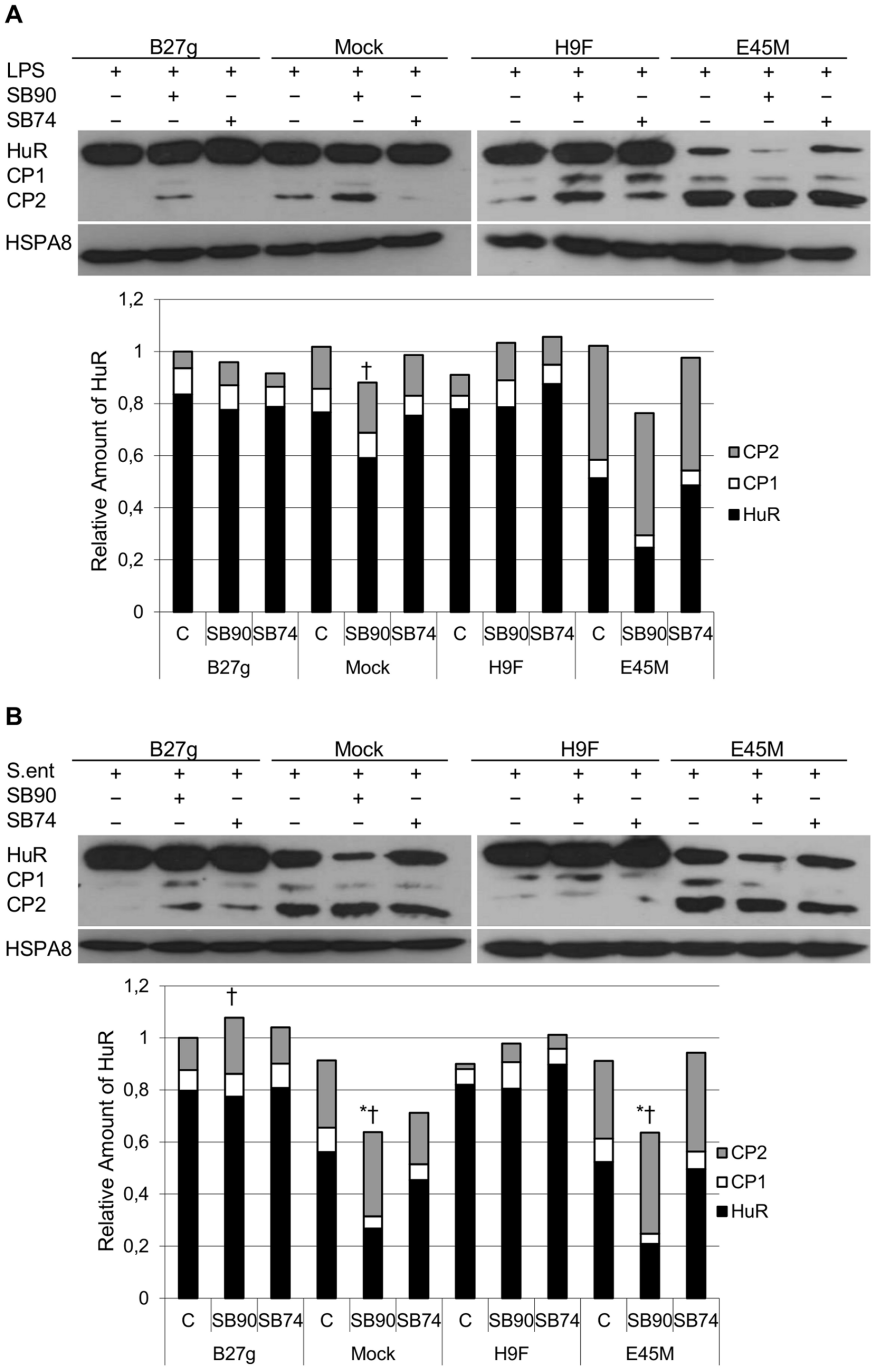
p38 inhibition effects on HuR expression and regulation in U937 monocytic cells. Full length HuR (36 kDa) and HuR cleavage products (CP1; 24 kDa and CP2; 8 kDa) in LPS-stimulated (A) and *Salmonella enteritidis*-infected (B) p38 inhibitor- or negative control for p38 inhibition-treated U937 Mock, B27g, H9F and E45M cells at 5 h time point (SB90; p38 inhibitor SB202190 and SB74; control for p38 inhibition SB202474) Expression of HuR was determined by SDS-PAGE and Western blotting. HSPA8 was used as a loading control. Results from densitometric analysis of five independent experiments are shown in diagram. See Materials and Methods for details. * = P-value ≤0.06 for HuR and ^†^ = P-value ≤0.06 for CP2 versus untreated sample at the respective cell line.

We have previously reported that p38 MAPK signaling is dysregulated in HLA-B27–transfected cells. It has also been shown that HuR is a direct substrate of p38 and LPS regulates its p38-dependent activation [20], [28]. Therefore we studied whether the expression of HuR is dependent on p38 activation after infection with *Salmonella* or LPS stimulation (Fig. 3). We inhibited p38 activity with a p38 inhibitor (SB202190). To ensure the inhibitor specificity, a control inhibitor (SB202474) was also used (apart from p38 inhibition, the control compound has the same influence on cell behavior as the actual inhibitor). In control cells that lack HLA-B27 expression (Mock) and in the cells expressing non-misfolding mutant of HLA-B27 (E45M) inhibition with SB202190 caused a decrease in the expression level of full length HuR. In *Salmonella*-infected cells (Fig. 3B), a decrease was observed (P-value ≤0.06) whereas in LPS-stimulated cells (Fig. 3A and B), the effect was similar but less profound (due to variation between the experiments no statistical difference was obtained). In contrast, when B27g and H9F cells were treated with the p38 inhibitor, HuR expression level remained unchangeable.

**Figure 4:**
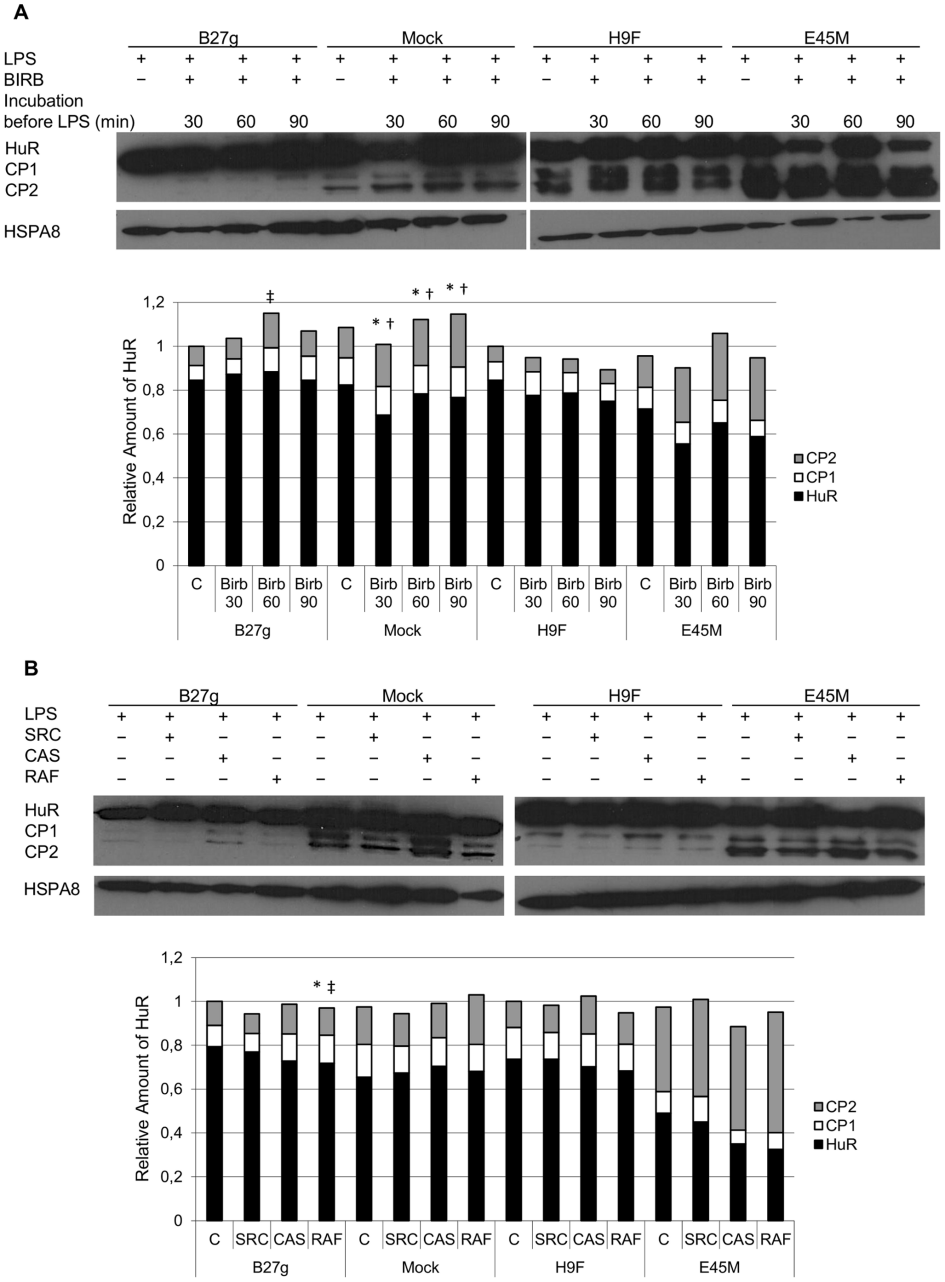
HuR expression was not affected by Src, Raf1 and CaseinI inhibition in U937 monocytic cells. Full length HuR (36 kDa) and HuR cleavage products (CP1; 24 kDa and CP2; 8 kDa) in LPS-stimulated U937 Mock, B27g, H9F and E45M cells at 5 h time point. In A, p38 inhibitor BIRB796 (Birb) was incubated with the cells 30, 60 or 90 min prior to LPS stimulation. In B, Src, Casein I (Cas) and Raf1 kinase inhibitors were incubated with the cells 15 min prior to LPS stimulation. Expression of HuR was determined by SDS-PAGE and Western blotting. HSPA8 was used as a loading control. Results from densitometric analysis of five independent experiments are shown in diagram. See Materials and Methods for details. * = P-value ≤0.06 for HuR, ^‡^ = P-value ≤0.06 for CP1 and ^†^ = P-value ≤0.06 for CP2 versus untreated sample at the respective cell line.

To further evaluate the role of p38 MAPK, another p38 MAPK inhibitor BIRB 796 was used. Since BIRB 796 is known to be a slow binder [29], incubation time was increased to 30, 60 and 90 min prior to LPS stimulation (Figure 4A). Decreased full length HuR expression was observed in Mock cells (P-value ≤0.06). Similar, but not significant effect was observed in E45M cells after 30 min incubation with BIRB 796. No effect on full length HuR expression in B27g or H9F cells was observed but amount of CP1 was increased in B27g cells after 60 min of BIRB 796 incubation prior to LPS stimulation. Increased amount of CP2 was observed after all BIRB 796 incubation times in Mock cells. Apart from p38 inhibition, SB202190 is known to affect Src, Casein I and Raf1 kinase activities [30]; to study the role of these kinases, inhibitors against these kinases were used (Figure 4B). None of these inhibitors had notable effect on full length HuR or CP expression in any cell line, despite the small but noteworthy (P-value ≤0.06) decrease of full length HuR and CP1 in B27g cells after Raf1 inhibition.

### TNFα Secretion is PKR-dependent and IL-10 Secretion is PKR and p38 MAPK Dependent in U937 Cells

**Figure 5:**
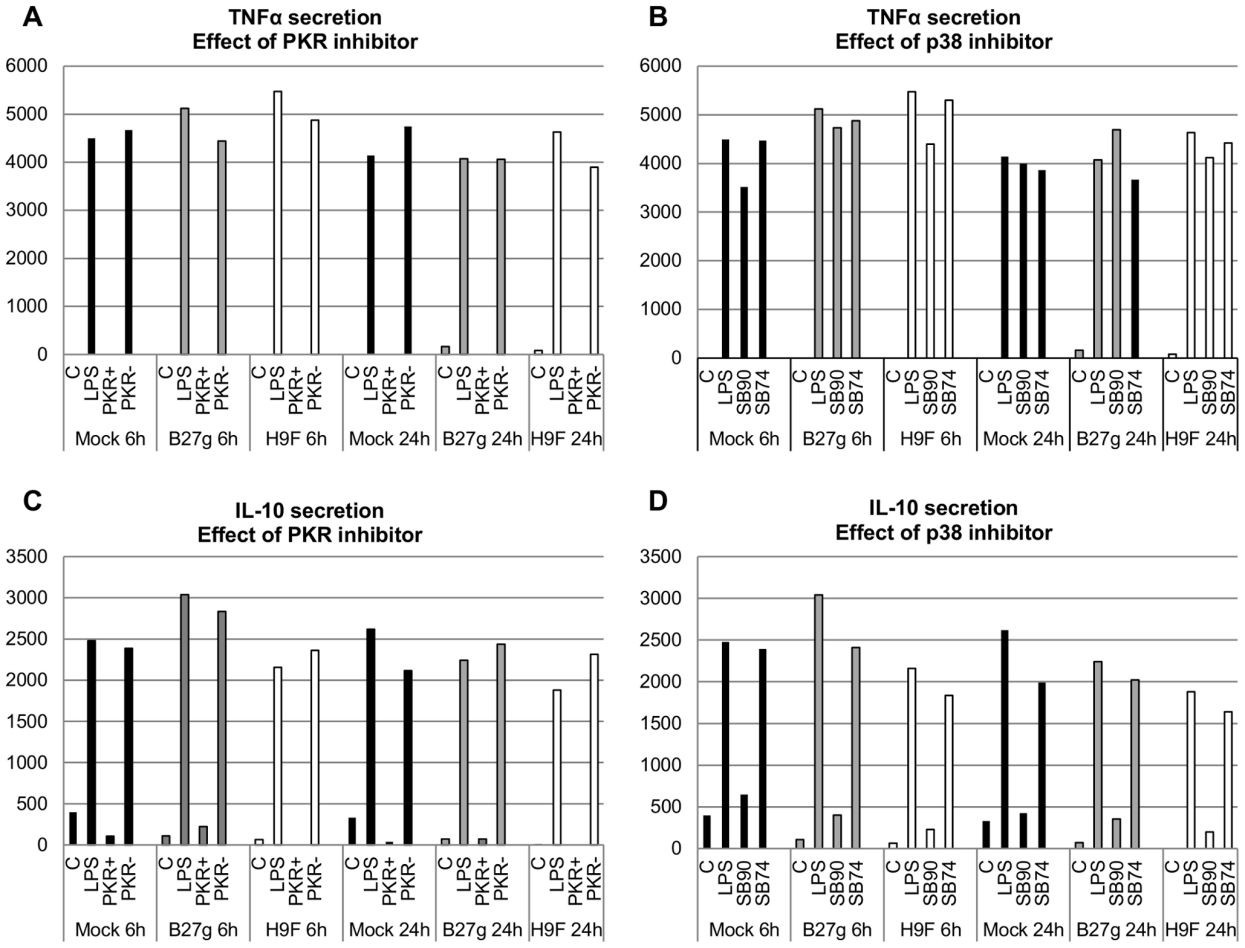
Secretion of TNFα and IL-10 in U937 monocytic cells is controlled by PKR and p38 MAPK. Cytokine secretion in Mock, B27g and H9F transfected U937 cells was determined 6 h and 24 h after LPS stimulation and treatment with PKR (PKR+) or p38 (SB90; p38 inhibitor SB202190) inhibitor. Controls for PKR and p38 inhibition were used (PKR- and SB74; control for p38 inhibition SB202474). TNFα secretion was measured after PKR inhibition in A and after p38 inhibition in B. IL-10 secretion was measured after PKR inhibition in C and after p38 inhibition in D. Values are the mean and SD of three or four independent experiments. See Materials and Methods for details.

There is evidence suggesting that HuR regulates TNFα expression [16]. Moreover, PKR and p38 MAPK are known to participate on TNFα and IL-10 production [31]–[36] and previously we observed that TNFα and IL-10 production are upregulated in B27g cells [37]. These findings prompted us to study whether HLA-B27 modulates p38- and/or PKR-dependent TNFα and IL-10 secretion in U937 cells. We used specific inhibitors for PKR (PKR+) and p38 (SB202190) and controls for inhibition (PKR- and SB202474, respectively). Similar to our earlier results [37], in all PMA-differentiated transfectants, no or negligible TNFα or IL-10 secretion was detected prior to LPS stimulation. All transfectants except E45M (these cells secreted low levels of TNFα and IL-10) secreted similar amounts of both cytokines 6 h and 24 h after LPS stimulation (Fig. 5A–D). p38 inhibition did not have substantial influence on TNFα secretion in any transfectants. In contrast, TNFα secretion was found to be completely dependent on PKR activity in all U937 transfectants since the inhibition of PKR caused total termination of TNFα secretion. Moreover, IL-10 secretion was found to be strongly both PKR- and p38–dependent in all transfectants as the amount of cytokine secreted after PKR- or p38- inhibition was similar to that observed prior to LPS stimulation.

## Discussion

Evidence exists suggesting that the interaction between ReA-triggering bacteria and HLA-B27 expressing host cell is abnormal [2], [4], [8], [26], [27], [38], [39]. We have previously observed that the presence of HLA-B27 HCs in monocytic cells modifies intracellular environment of U937 monocyte/macrophages in favor of the disease-triggering bacteria and we found that the regulation of several signaling molecules was altered in these cells [1]–[3], [26]. In this study, we show that the generation of HuR and its cleavage products (CP1 and CP2) are altered in monocytic cells expressing HLA-B27 HCs. Moreover, in these cells, HuR cleavage shows reduced dependence on PKR activity after *Salmonella* infection. These findings correlate with the expression of glutamic acid 45 in the B pocket that is linked to the misfolding of HLA-B27.

Control cells (Mock and E45M) generate substantially more CP1 and CP2 compared to misfolding HLA-B27-expressing cells (B27g and H9F) even prior to LPS stimulation or *Salmonella* infection. These results suggest that the activity of regulatory mechanism controlling HuR cleavage is more active in Mock and E45M cells. Recent evidence indicates that PKR is a major kinase regulating cleavage of HuR [23]. We have observed that in HLA-B27-expressing cells PKR is overexpressed and hypophosphorylated [3]. Thus, our results suggest that altered PKR regulation in cells expressing misfolding HLA-B27 HCs has functional consequences by altering the activity of PKR-dependent cleavage of HuR. This idea is further supported by studies using PKR inhibitor. In control cells, we observed increase in full length HuR and decrease in CP2 expression suggesting that PKR inhibition inhibits HuR cleavage to CP2 leading to accumulation of full length HuR (increased expression). In contrast, a substantial increase of full length HuR was not observed in B27g and H9F cells suggesting that PKR inhibition had a less dramatic effect on HuR cleavage in these cells. However, generation of CP1 is increased in all transfectants after PKR inhibition. We also used a control inhibitor for PKR inhibition. Since this control compound had only minor effect on HuR expression, we suggest that the effect observed on HuR expression after incubation with PKR inhibitor is at least mainly caused by PKR inhibition. Thus these results suggest that PKR dependent HuR regulation is already disturbed in HLA-B27 expressing cells. HuR cleavage to CP1 and CP2 is an important mechanism to control the regulation of apoptosis. Therefore, these modulatory effects may have an important role regulating cell fate. In fact, our previous results suggest that cell death may be altered in HLA-B27-expressing U937 cells [3].

According to literature, another important regulator of HuR is p38 MAPK [28], [40]. p38 activation causes a rapid cytoplasmic accumulation of HuR [40]. Interestingly, we have seen that p38–related signaling is dysregulated in HLA-B27 positive cells [2], [3]. Therefore, we aimed to study whether p38 inhibition influences on the expression of full length HuR and its cleavage products CP1 and CP2, and whether HLA-B27 expression modulates p38-dependent HuR regulation. Interestingly, we observed that p38 inhibition by SB202190 failed to downregulate full length HuR in HLA-B27-expressing cells, whereas a decrease in HuR expression and increase in CPs was observed in control cells. We used a negative control inhibitor similar to SB202190 to ensure the specificity of the p38 inhibition. This compound had only a minor effect on HuR expression, suggesting that changes observed on HuR expression after incubation with SB202190 are caused by p38 inhibition. To further study the role of p38 activity on HuR expression another p38 inhibitor BIRB 796 was used. Preliminary experiments were performed with similar protocol as used with other inhibitors. In contrast to results observed with SB202190, no effect on HuR expression after 5 hours of LPS stimulation was observed. This might be due to methodological limitations, since BIRB 796 binds slowly to its target [29]. For that reason, we increased the incubation time, and after that we were able to see a similar, but less profound effect on HuR expression than observed after incubation with SB202190.

Since the effect observed in HuR expression after inhibition with BIRB 796 was less dramatic, the role of other kinases affected by SB202190 was studied using chemical inhibitory compounds [30]. Src kinase inhibitor is known to reduce receptor-interacting serine-threonine kinase 2 (RIPK2) activity while Raf inhibitor reduces cyclin G associated kinase (GAK) activity [30]. Casein inhibitor is targeted against casein kinase I delta (CSNK1D) [30]. After Src, Casein I and Raf1 inhibitors were used, no effect on HuR expression was observed. Only in B27g-transfected cells, expression levels of full length HuR and CP1 decreased after treatment with Raf inhibitor. However, if the effect observed after SB202190 treatment would be GAK dependent, decreased HuR expression should have been observed in B27g cells also after incubation with SB202190. In conclusion, our findings do not rule out the involvement of other kinases than p38 MAPK in HuR regulation but the findings obtained by using SB202190 and BIRB 796 support the idea that p38 is of importance. Further studies are required to solve these questions. However, a possible defect to downregulate HuR expression by inhibiting p38 activity observed in HLA-B27-expressing cells may have severe consequences. For example, IL-10, a potent anti-inflammatory cytokine suppresses p38 activation and HuR expression in U937 cells [41], [42]. Thus, failure to downregulate HuR expression through p38 inhibition observed in HLA-B27-expressing transfectants may prevent IL-10 to mediate some of its anti-inflammatory functions (e.g. downregulate TNFα secretion).

Altered regulation of HuR in HLA-B27 expressing U937 cells prompted us to study whether LPS-induced PKR- and p38-dependent regulation of IL-10 and/or TNFα secretion is modulated in these cells. Results indicate that in U937 transfectants, PKR plays indispensable role in TNFα secretion (Fig. 5A). This is in line with previous observations showing that PKR activation by phosphorylation is critically involved in the secretion of TNFα [31]. In contrast, p38 inhibition by SB202190 did not impact significantly on the amount of secreted TNFα in U937 cells. Thus, it is unlikely that HuR or expression of HLA-B27 HCs play a major role in TNFα secretion in U937 monocytic cells, since although p38 inhibition remarkably decreased HuR expression in Mock transfectants (Fig. 3A and C), TNFα secretion was not altered by p38 inhibition or expression of HLA-B27 HCs (Fig. 5B). Contrary to TNFα secretion, both PKR- and p38-inhibition dramatically decreased IL-10 secretion in all studied cell lines. However, it is not likely that defect in IL-10 regulation would explain altered HuR expression or CP generation in HLA-B27 expressing cells, since secretion of the IL-10 was similarly controlled by PKR and p38 both in HLA-B27-expressing and control cells.

In addition to TNFα and IL-10, HuR regulates also many other important genes. For example, C/EBPβ is a transcription factor involved in the regulation of inflammatory response. It also regulates *Salmonella* survival in mouse macrophages [43]. Our previous results show that C/EBPβ expression upon LPS-stimulation is PKR-dependent in U937 cells [3]. However, in HLA-B27–expressing U937 cells, it is overexpressed and mainly PKR-independent [3]. This could be explained by HuR (36 kDa) overexpression. HuR enhances C/EBPβ mRNA stability and translation, leading to increased amount of the protein [18]. Controversial findings where HuR binding decreases the C/EBPβ expression exist, HuR binding to C/EBPβ mRNA is suggested to diminish its movement to cytosol [22]. Thus it is also possible that the regulation of HuR binding is disturbed in HLA-B27 cells and this leads to increased cytoplasmic C/EBPβ mRNA and protein production and results in C/EBPβ overexpression. Taken into account this, it would be of interest to investigate C/EBPβ expression in HLA-B27-expressing cells after HuR downregulation. Unfortunately, HuR siRNA did not work in our hands and further experiments are needed.

HuR is involved in apoptosis by regulating both pro- and antiapoptotic mRNAs [21], [23], [44], [45]. In response to severe extracellular stress, HuR is cleaved to CP1 and CP2. In fact, without being activated by phosphorylation PKR activates the caspase-8/caspase-3 pathway to trigger HuR cleavage, and the CPs are then capable to promote apoptosis [23]. Previously we observed that in HLA-B27-expressing monocytic U937 cells PKR is overexpressed but hypophosphorylated [3]. In response to increased PKR expression, higher HuR cleavage rate in HLA-B27 cells was expected. However, here we observed increased HuR (36 kDa) expression but decreased HuR CP expression in these cells and we conclude that HuR cleavage by PKR is impaired in HLA-B27 cells. Cell death is delayed without HuR cleavage [21], [23] and PKR regulates *Salmonella*-induced cell death in a p38-dependent manner [46]. It is possible that modifications on p38 and PKR regulation in HLA-B27–expressing U937 cells modulate intracellular *Salmonella* survival and that these modifications in signaling generate a more favorable intracellular environment for pathogen survival and replication [3]. Also decreased HuR cleavage in response to LPS-stimulation and *Salmonella*-infection implies that HLA-B27–expressing cells are more resistant to cell death. This conclusion is supported by our finding that LPS-stimulated HLA-B27–expressing U937 cells are more tolerant to cell death [3].

The exact mechanism of the role that HLA-B27 and its misfolding HCs have in the cellular signaling remains to be achieved. In this study, we provide evidence that the expression of HLA-B27 in U937 modifies the regulation of HuR. We also show that the effect of HLA-B27 on HuR regulation is dependent on E45 amino acid residue in the peptide binding groove that is at least partially responsible for the misfolding feature of the HLA-B27 molecule. Since HuR is a central regulator of the inflammatory response, our findings provide a novel mechanism by which HLA-B27 modulates the interaction between host cell and ReA triggering bacteria.
